# Stability and interpretability of penalized logistic regression models for breast cancer risk prediction

**DOI:** 10.1371/journal.pone.0353489

**Published:** 2026-07-28

**Authors:** Francis Okyere, Michael Nyanney

**Affiliations:** Department of Statistics, Florida State University, Tallahassee, Florida, United States of America; University of Mosul, IRAQ

## Abstract

Penalized logistic regression is widely used in biomedical classification to address multicollinearity and improve predictive performance, yet the stability and reproducibility of selected predictors are often overlooked. This study evaluates feature stability and interpretability in ridge, lasso, and elastic-net logistic regression for breast cancer diagnosis using the Wisconsin Diagnostic Breast Cancer dataset. Models were trained with cross-validated tuning and evaluated on an independent test set using discrimination, classification, and calibration metrics. Feature stability was quantified through bootstrap selection frequencies. All penalized models achieved near-perfect discrimination and improved calibration compared with unpenalized logistic regression. However, substantial differences emerged in stability and sparsity. Ridge regression exhibited maximal stability but retained all predictors, limiting interpretability. Lasso regression produced highly sparse models but showed greater selection variability. Elastic-net regression balanced sparsity and stability, consistently retaining correlated predictors linked to tumor morphology. These findings demonstrate that stability assessment provides critical information beyond predictive accuracy and supports stability-aware penalized modeling for interpretable and reproducible biomedical risk prediction.

## 1. Introduction

Accurate and reliable statistical models play a critical role in medical diagnosis and risk prediction, particularly in oncology, where early detection substantially improves patient outcomes. Breast cancer diagnosis based on quantitative imaging and cytological measurements has become a standard benchmark problem for evaluating statistical and machine learning methods. While predictive accuracy is essential, clinical deployment of diagnostic models also requires interpretability, reproducibility, and stability of selected predictors, as unstable models can undermine trust and hinder scientific understanding [1]. Consequently, resampling-based stability assessment has become an increasingly important component of interpretable machine-learning pipelines in biomedical research [2]. The Wisconsin Diagnostic Breast Cancer dataset provides a well-studied benchmark with strong predictor correlations, making it particularly suitable for evaluating stability and interpretability in penalized models.

Logistic regression has long been a cornerstone of medical risk modeling due to its probabilistic interpretation and transparent coefficient structure [1]. However, modern biomedical datasets frequently contain large numbers of correlated predictors derived from related biological or imaging processes. In such settings, maximum likelihood estimation can yield unstable coefficient estimates with inflated variance, motivating the widespread adoption of penalized regression techniques.

Penalized logistic regression methods, including ridge regression [3], the lasso [4], and the elastic-net [5], address these challenges by introducing regularization terms that control model complexity. Ridge regression stabilizes coefficient estimates through shrinkage, while the lasso performs variable selection by enforcing sparsity. Elastic-net regression combines these penalties to accommodate groups of correlated predictors while retaining sparsity. These methods have been successfully applied across numerous biomedical classification problems and are frequently reported to achieve strong predictive performance.

Despite their popularity, variable selection results from penalized models are often treated as definitive, even though they may be highly sensitive to sampling variability, tuning parameter choices, and minor perturbations of the data. In biomedical contexts, where predictors are often correlated and sample sizes are moderate, this sensitivity can lead to inconsistent identification of important variables across repeated analyses. As a result, interpretability claims based solely on a single fitted penalized model may be misleading [6,7].

This concern has motivated growing interest in feature stability, defined as the reproducibility of variable selection under data resampling or perturbation. Stability is closely linked to interpretability: predictors that are consistently selected across resamples are more likely to reflect genuine signal rather than artifacts of random variation. Meinshausen and Bühlmann formalized this concept through stability selection, demonstrating that resampling-based approaches can substantially improve the reliability of variable selection procedures [8]. Subsequent studies have emphasized that stability assessment is essential for reproducible scientific inference, particularly in settings involving correlated or high-dimensional predictors [6,7].

In medical prediction modeling, stability considerations are especially important. Diagnostic models are often used to inform clinical decisions, and unstable predictor sets can hinder interpretability, limit generalizability, and reduce clinician confidence. While prior studies have compared penalized regression methods in terms of predictive accuracy, fewer have systematically evaluated how stability differs across penalties and how these differences affect interpretability in applied medical settings.

The objective of this study is to investigate feature stability and interpretability in penalized logistic regression, with particular emphasis on comparing ridge, lasso, and elastic-net penalties. We propose a resampling-based framework that quantifies feature stability using bootstrap selection frequencies, enabling identification of predictors that are robust to sampling variability. Predictive performance and calibration are evaluated alongside stability measures to illustrate trade-offs between sparsity, robustness, and interpretability.

This study makes three practical contributions to stability-aware clinical modeling. First, we present a reproducible interpretability workflow for penalized logistic regression that integrates coefficient path analysis, bootstrap selection frequencies, and complementary performance metrics to assess predictor reliability. Second, we provide a systematic empirical comparison of ridge, lasso, and elastic-net regression in breast cancer diagnosis, highlighting trade-offs between predictive accuracy, feature stability, and model parsimony. Third, we demonstrate how stability diagnostics can inform trustworthy biomarker selection and support deployment of penalized models in clinical decision-support systems. Collectively, these contributions emphasize the importance of stability-aware evaluation for reliable medical risk prediction and reproducible biomedical research.

## 2. Data Description

### 2.1. Data source

The data used in this study are publicly available from the Breast Cancer Wisconsin (Diagnostic) Dataset [9]. This dataset compiles quantitative measurements derived from digitized images of fine-needle aspirates (FNA) of breast masses and has been widely used for benchmarking statistical and machine learning methods in biomedical classification. All analyses reported in this manuscript are based on the version of the dataset downloaded on December 15, 2025. The dataset has been extensively used as a benchmark in statistical learning and medical classification studies, allowing direct comparison with prior methodological evaluations.

### 2.2. Outcome variable

The primary response variable is diagnosis, a categorical indicator of tumor status with levels benign (B) and malignant (M). For modeling purposes, the outcome was re-encoded as a binary response:


$$\begin{array}{c} Y_{i}=\left\{ \begin{array}{c} @l@1,\text{\textbackslash{}hspace\{0.17em}\;\}malignant\left(M\right)\\ 0,\text{\textbackslash{}hspace\{0.17em}\;\}benign\left(B\right)\text{\textbackslash{}hspace\{0.17em}\} \end{array}\right. \end{array}$$

Logistic regression and its penalized variants were used to estimate the probability of malignancy.

### 2.3. Predictor variables

The dataset includes 30 continuous predictor variables describing geometric and textural properties of cell nuclei, originally derived from FNA images. These features quantify tumor morphology and texture characteristics that are relevant for diagnostic classification [6]. Predictors fall into the following categories:

- Size-related measures: radius, perimeter, and area
- Shape-related measures: concavity, concave points, symmetry, and compactness
- Texture-related measures: texture, smoothness, and related statistics

For each of these characteristics, three summary values are provided: the mean, the standard error (SE), and the worst (maximum) observed value across tumor cells. This hierarchical structure results in groups of strongly correlated features that reflect redundant and related biological information.

### 2.4. Data preprocessing

Prior to analysis, non-informative variables, such as patient identifiers, were excluded. An additional column that contained only missing values was also removed. The remaining predictors contained no missing observations and therefore required no imputation. All continuous predictors were standardized to zero mean and unit variance using parameters estimated only from the training data. Standardization ensures that penalization penalties are applied equitably across predictors, preventing differences in scale from driving variable selection or coefficient shrinkage.

### 2.5. Training and test splits

To assess out-of-sample performance and avoid optimistic bias, the dataset was randomly partitioned into training (80%) and test (20%) subsets using stratified sampling to preserve the class distribution. All model fitting, tuning penalization parameters, and stability assessment were performed exclusively on the training data. Final performance evaluation, including discrimination and calibration metrics, was conducted on the held-out test set.

### 2.6. Motivation for penalization and stability assessment

The strong correlations among predictors and the moderate sample size, relative to the number of features, indicate that standard unpenalized logistic regression may yield unstable coefficient estimates. Penalized regression methods like ridge, lasso, and elastic-net are therefore well-suited for this setting. However, penalization alone does not guarantee reproducible or interpretable variable selection; hence, a resampling-based feature stability analysis is incorporated to identify predictors that consistently contribute to diagnostic classification across data perturbations.

## 3. Methodology

This section describes the statistical modeling framework, feature stability assessment procedures, and evaluation strategy used to compare penalized logistic regression models. The methodology is designed to support reproducible assessment of predictive performance, calibration, and feature stability while avoiding optimistic bias through strict separation of training, tuning, and test evaluation. All analyses follow established best practices for clinical prediction modeling and stability assessment [1,8,10].

### 3.1. Logistic regression framework

Let $\left(x_{i},y_{i}\right)$ for i=1,...,n denote independent observations, where $x_{i}={\left(x_{i1},x_{i2},...,x_{ip}\right)}^{T}$ is a vector of standardized predictors and $y_{i}\in \left\{0,1\right\}$ is a binary response variable indicating tumor status, with $y_{i}=1$ corresponding to malignant tumors and $y_{i}=0$ corresponding to benign tumors.

Logistic regression models the conditional probability of malignancy as


$$\begin{array}{c} P\left(Y_{i}=1|x_{i}\right)=\pi_{i}=\frac{exp\left(\beta_{\text{0}}+x_{i}^{T}\beta \right)}{1+exp\left(\beta_{\text{0}}+x_{i}^{T}\beta \right)} \end{array}$$ (1)

where $\beta_{\text{0}}$ is an intercept and $\beta ={\left(\beta_{\text{1}},\beta_{\text{2}},...,\beta_{p}\right)}^{T}$ is the vector of regression coefficients.

Equivalently, the log-odds (logit) relationship is given by


$$\begin{array}{c} log\left(\frac{\pi_{i}}{1-\pi_{i}}\right)=\beta_{\text{0}}+x_{i}^{T}\beta \end{array}$$

Assuming conditional independence of observations given the predictors, each response $Y_{i}$ follows a Bernoulli distribution with success probability $\pi_{i}$. The Bernoulli probability mass function is therefore


$$\begin{array}{c} P\left(Y_{i}=y_{i}|x_{i}\right)=\pi_{i}^{y_{i}}{\left(1-\pi_{i}\right)}^{1-y_{i}} \end{array}$$

Taking the product over all observations yields likelihood function


$$\begin{array}{c} L\left(\beta_{\text{0}},\beta \right)=\prod_{i=1}^{n}\pi_{i}^{y_{i}}{\left(1-\pi_{i}\right)}^{1-y_{i}}. \end{array}$$

Applying the natural logarithm gives the log-likelihood:


$$\begin{array}{c} \ell \left(\beta_{\text{0}},\beta \right)=\sum_{i=1}^{n}\left[y_{i}log\left(\pi_{i}\right)+\left(1-y_{i}\right)log\left(1-\pi_{i}\right)\right]. \end{array}$$ (2)

Equation (2) clarifies the role of the response variable $y_{i}$: when $y_{i}=1$, the malignant probability term contributes to the likelihood; when $y_{i}=0$, the benign probability term contributes instead. This representation forms the basis for both classical maximum likelihood estimation and penalized extensions. Under standard regularity conditions, maximum likelihood estimators are consistent and asymptotically normal. However, in the presence of strong predictor correlations, coefficient estimates may exhibit inflated variance, instability, and reduced interpretability, motivating penalized extensions of logistic regression [3–6].

### 3.2. Penalized logistic regression

Penalized logistic regression estimates model parameters by maximizing a penalized version of the log-likelihood, equivalently minimizing the negative penalized log-likelihood:


$$\begin{array}{c} \hat{\beta }=\underset{\beta_{\text{0}},\beta }{argmax}\left\{\ell \left(\beta_{\text{0}},\beta \right)-\lambda P\left(\beta \right)\right\} \end{array}$$ (3)

where P(β) is a penalty function and λ≥0 controls the strength of regularization.

Penalization introduces bias but reduces estimator variance, often improving predictive stability and out-of-sample performance in finite samples [6].

#### 3.2.1. Ridge penalty.

The ridge regression, the penalty function is


$$\begin{array}{c} P\left(\beta \right)=\sum_{j=1}^{p}\beta_{j}^{\text{2}}={\|\beta \|}_{\text{2}}^{\text{2}} \end{array}$$ (4)

Thus, ridge logistic regression solves


$$\begin{array}{c} {\hat{\beta }}_{ridge}=\underset{\beta_{\text{0}},\beta }{argmax}\left\{\ell \left(\beta_{\text{0}},\beta \right)-\lambda \sum_{j=1}^{p}\beta_{j}^{\text{2}}\right\} \end{array}$$

Ridge regression continuously shrinks coefficients toward zero without setting them exactly to zero. Because the objective function is strictly convex for λ>0, ridge regression yields a unique solution and is particularly effective for stabilizing coefficient estimation under multicollinearity [3].

#### 3.2.2. Lasso penalty.

For lasso regression, the penalty is defined as


$$\begin{array}{c} P\left(\beta \right)=\sum_{j=1}^{p}\left|\beta_{j}\right|={\|\beta \|}_{\text{1}} \end{array}$$ (5)

The lasso estimator is therefore


$$\begin{array}{c} {\hat{\beta }}_{lasso}=\underset{\beta_{\text{0}},\beta }{argmax}\left\{\ell \left(\beta_{\text{0}},\beta \right)-\lambda \sum_{j=1}^{p}\left|\beta_{j}\right|\right\}. \end{array}$$

Unlike ridge regression, the $\ell_{\text{1}}$ penalty is non-differentiable at zero, which induces sparsity by shrinking some coefficients exactly to zero, thereby enabling simultaneous estimation and variable selection [4]

### Sparsity property (informal justification)

From the Karush-Kuhn-Tucker optimality conditions, a coefficient is set exactly to zero whenever the absolute score contribution is sufficiently small relative to the penalty parameter λ. Consequently, the lasso creates a thresholding region around zero in coefficient space, yielding sparse models with improved interpretability.

#### 3.2.3. Elastic-net penalty.

The elastic-net penalty combines $\ell_{\text{1}}$ and $\ell_{\text{2}}$ regularization:


$$\begin{array}{c} P\left(\beta \right)=\alpha \sum_{j=1}^{p}\left|\beta_{j}\right|+\left(1-\alpha \right)\sum_{j=1}^{p}\beta_{j}^{\text{2}},\text{\textbackslash{}hspace\{0.17em}\;\;\;\;\;\}0\leq \alpha \leq 1. \end{array}$$ (6)

The elastic-net estimator becomes


$$\begin{array}{c} {\hat{\beta }}_{EN}=\underset{\beta_{\text{0}},\beta }{argmax}\left\{\ell \left(\beta_{\text{0}},\beta \right)-\lambda \left[\alpha \sum_{j=1}^{p}\left|\beta_{j}\right|+\left(1-\alpha \right)\sum_{j=1}^{p}\beta_{j}^{\text{2}}\right]\right\}. \end{array}$$

where α=1, elastic-net reduces to lasso regression; α=0, it reduces to ridge regression.

Intermediate values combine sparsity with grouped selection of correlated predictors, addressing limitations of pure lasso penalization in correlated biomedical data [5].

### 3.3. Theoretical considerations

#### 3.3.1. Convexity and solution properties.

For all penalties considered, the penalized objective function remains convex. Ridge and elastic-net penalties yield strictly convex objectives for λ>0, ensuring unique solutions. While the lasso objective may admit multiple coefficient vectors in some high-dimensional settings, fitted values remain unique under mild regularity conditions [7].

#### 3.3.2. Bias-Variance Trade-off.

Penalization introduces estimation bias but reduces variance, often lowering overall prediction error. Ridge and elastic-net penalties are particularly effective when predictors are strongly correlated, whereas lasso prioritizes sparsity. These properties motivate comparison of penalization strategies in terms of trade-offs among predictive accuracy, sparsity, stability, and interpretability [6].

### 3.4. Hyperparameter selection

The regularization parameter λ and the elastic-net mixing parameter α were selected using stratified 10-fold cross-validation on the training data. Optimal tuning parameters minimizing the cross-validated binomial deviance. Hyperparameter tuning was conducted independently of both stability analysis and final test-set evaluation to avoid optimistic bias.

For additional discussion of ridge regression, lasso, and elastic-net estimation in linear and logistic regression settings, we refer readers to [11,12], among others.

### 3.5. Feature stability analysis

#### 3.5.1. Definition of feature stability.

Let $S^{\left(b\right)}$ denote the set of predictors selected in bootstrap sample b for b=1,2,...,B. The selection frequency of predictor j is defined as


$$\begin{array}{c} {\hat{\pi }}_{j}=\frac{\text{1}}{B}\sum_{b=1}^{B}I\left\{j\in S^{\left(b\right)}\right\}, \end{array}$$ (7)

where I(·) denotes the indicator function.

A predictor is considered stable when its selection frequency exceeds a predefined threshold $\pi^{*}$. In this study, we use $\pi^{*}=0.70$, consistent with common practice in stability selection [8].

#### 3.5.2. Bootstrap stability procedure.

Feature stability was assessed as follows:

1. Draw B bootstrap samples from the training data.
2. Fit the penalized logistic regression model using fixed tuning parameters.
3. Record the set of selected predictors for each bootstrap sample.
4. Compute bootstrap selection frequencies using Equation (7).

A bootstrap size of B=1000 was used to provide a stable approximation of selection frequencies while reducing Monte Carlo variability in the estimated stability measures. This larger resampling size improves precision of stability estimates while remaining computationally feasible for the present analysis.

#### 3.5.3. Interpretation and theoretical motivation.

Stability selection provides a finite-sample measure of reproducibility for variable selection procedures. Predictors consistently selected across resampled datasets are more likely to represent robust signal rather than artifacts of sampling variability, noise, or multicollinearity [8].

### 3.6. Model evaluation strategy

To ensure an unbiased assessment of predictive performance, the dataset is partitioned into training and test subsets. Model fitting, tuning, and stability analysis are conducted exclusively on the training data. Final performance metrics, including accuracy, sensitivity, specificity, area under the ROC curve, and calibration measures, are computed on the held-out test data.

This separation allows feature stability to be interpreted independently of predictive evaluation and reflects recommended practices in medical prediction modeling [1].

### 3.7. Ethics statement

This study used the publicly available Breast Cancer Wisconsin (Diagnostic) dataset obtained from the University of California, Irvine Machine Learning Repository, available at https://archive.ics.uci.edu/dataset/17/breast±cancer±wisconsin±diagnostic. The dataset was accessed through its Kaggle mirror (Kaggle) for analysis. It is fully anonymized, publicly accessible, and contains no personally identifiable information. Therefore, institutional review board (IRB) approval and informed consent were not required because this study involved secondary analysis of de-identified, publicly available data.

### 3.8. Reproducibility and software

All analyses were conducted in R using publicly available packages, including glmnet,\hspace{0.17em}pROC,\hspace{0.17em}rsample,\hspace{0.17em}yardstick and tidyverse. Code used for preprocessing, model fitting, bootstrap stability analysis, and figure generation will be made publicly available upon publication to support reproducibility and reuse.

## 4. Evaluation Metrics

Evaluation of diagnostic prediction models in medical research requires assessment of multiple complementary dimensions of performance, including discrimination, classification accuracy at clinically relevant thresholds, probability calibration, and interpretability. Accordingly, model performance in this study is evaluated using a combination of classification-based, threshold-independent, calibration, and stability metrics, all computed on an independent held-out test set.

### 4.1. Classification performance metrics

Classification performance is summarized using the confusion matrix, which comprises true positives (TP), true negatives (TN), false positives (FP), and false negatives (FN) [1]. These quantities provide the basis for threshold-dependent performance measures that are directly relevant to clinical decision-making.

#### 4.1.1. Accuracy.


$$\begin{array}{c} Accuracy=\frac{TP+TN}{TP+TN+FP+FN} \end{array}$$ (8)

Accuracy represents the proportion of correctly classified observations across both malignant and benign cases.

#### 4.1.2. Sensitivity and Specificity.

Sensitivity (true positive rate) is defined as


$$\begin{array}{c} Sensitivity=\frac{TP}{TP+FN} \end{array}$$ (9)

and measures the ability of the model to correctly identify malignant cases. Specificity (true negative rate) is defined as


$$\begin{array}{c} Specificity=\frac{TN}{TN+FP} \end{array}$$ (10)

and reflects the ability to correctly identify benign cases. Sensitivity and specificity are reported jointly to capture the asymmetric clinical consequences of false-negative and false-positive diagnostic errors.

### 4.2. Threshold selection

Predicted probabilities are converted into binary class labels using two thresholding strategies:

1. A fixed threshold of 0.5, which provides a standard reference point.
2. An optimal threshold selected via Youden’s index, defined as

$$\begin{array}{c} J=Sensitivity+Specificity-1 \end{array}$$ (11)

which maximizes the combined sensitivity and specificity [13].

Reporting results under both thresholds allows assessment of model robustness to threshold choice and facilitates interpretable comparisons across models.

### 4.3. Discriminative ability

Discriminative performance is assessed using the area under the receiver operating characteristic curve (AUC). The ROC curve plots sensitivity against the false positive rate across all possible classification thresholds. The AUC represents the probability that the model assigns a higher predicted risk to a randomly selected malignant case than to a randomly selected benign case. Values range from 0.5 (no discriminative ability) to 1 (perfect discrimination). Because the AUC is threshold-independent, it is well suited for comparing models with different probability distributions and classification thresholds [14].

### 4.4. Calibration and probability accuracy

While discrimination evaluates ranking ability, calibration assesses the agreement between predicted probabilities and observed outcomes. Calibration is particularly important in medical risk prediction, where probabilities may inform downstream clinical decisions.

Two complementary measures are used:

#### 4.4.1. Brier score.


$$\begin{array}{c} Brier=\frac{\text{1}}{n}\sum_{i=1}^{n}{\left({\hat{p}}_{i}-y_{i}\right)}^{\text{2}} \end{array}$$ (12)

where ${\hat{p}}_{i}$ denote the predicted probability for observation i and $y_{i}$ is the corresponding binary outcome. Lower Brier scores indicate better probability accuracy.

#### 4.4.2. Calibration plots.

Calibration plots are constructed by grouping predicted probabilities into deciles and plotting observed event rates against mean predicted probabilities within each group. Deviations from the 45-degree reference line indicate miscalibration.

Together these measures assess whether strong discrimination is accompanied by reliable and well-calibrated probability estimates [1].

### 4.5. Stability metrics

To evaluate reproducibility of variable selection, feature stability is quantified using bootstrap selection frequencies. For each predictor, stability is defined as the proportion of bootstrap resamples in which the predictor is selected.

Stability metrics complement predictive performance measures by addressing robustness of interpretation. Models that achieve high predictive accuracy but exhibit unstable variable selection may be less suitable for scientific inference or clinical interpretation.

### 4.6. Model comparison strategy

All evaluation metrics are computed on a held-out test set that is not used for model fitting, tuning, or stability estimation. Models are compared jointly across discrimination, classification performance, calibration, and stability metrics to characterize trade-offs between predictive accuracy, sparsity, and interpretability. This multi-criteria evaluation framework reflects recommended practices in medical prediction modeling and avoids reliance on a single performance measure [1].

## 5. Results

### 5.1. Predictive performance of penalized and unpenalized models

Table 1 summarizes the predictive performance of the unpenalized logistic regression model and the penalized logistic regression models (ridge, lasso, and elastic-net), evaluated on the held-out test set using Youden’s optimal threshold. Across all modeling approaches, discriminative performance is uniformly high, reflecting the strong signal present in the data. All penalized models achieve an accuracy of 0.991 with AUC values approaching unity, whereas the unpenalized logistic regression model attains a slightly lower accuracy of 0.965.

**Table 1:** Predictive performance of unpenalized logistic regression, ridge, lasso, and elastic-net models on the test set using Youden’s optimal threshold (accuracy, sensitivity, specificity, AUC, and Brier score).

model	threshold	accuracy	sensitivity	specificity	auc	brier	tp	tn	fp	fn
Ridge	0.459	0.991	0.977	1.000	0.999	0.021	42	72	0	1
Lasso	0.480	0.991	1.000	0.986	0.999	0.015	43	71	1	0
Elastic-Net	0.522	0.991	1.000	0.986	0.999	0.012	43	71	1	0
GLM	0.000	0.965	1.000	0.944	0.972	0.043	43	68	4	0

Despite comparable overall discrimination, differences emerge in error structure and probability accuracy. The unpenalized model achieves perfect sensitivity but exhibits reduced specificity (0.944), resulting in a higher false-positive rate. In contrast, ridge regression attains perfect specificity with a single false negative, while lasso and elastic-net models achieve perfect sensitivity with one false positive each. These patterns indicate that penalization improves the balance between sensitivity and specificity at clinically relevant decision thresholds.

Calibration performance, assessed using the Brier score, further differentiates the modeling approaches. Penalized models consistently outperform the unpenalized logistic regression model, with elastic-net regression yielding the lowest Brier score (0.012), followed closely by lasso and ridge regression. These results indicate improved probability accuracy and reduced overconfidence in predicted risk estimates under penalization.

**Fig 1:**
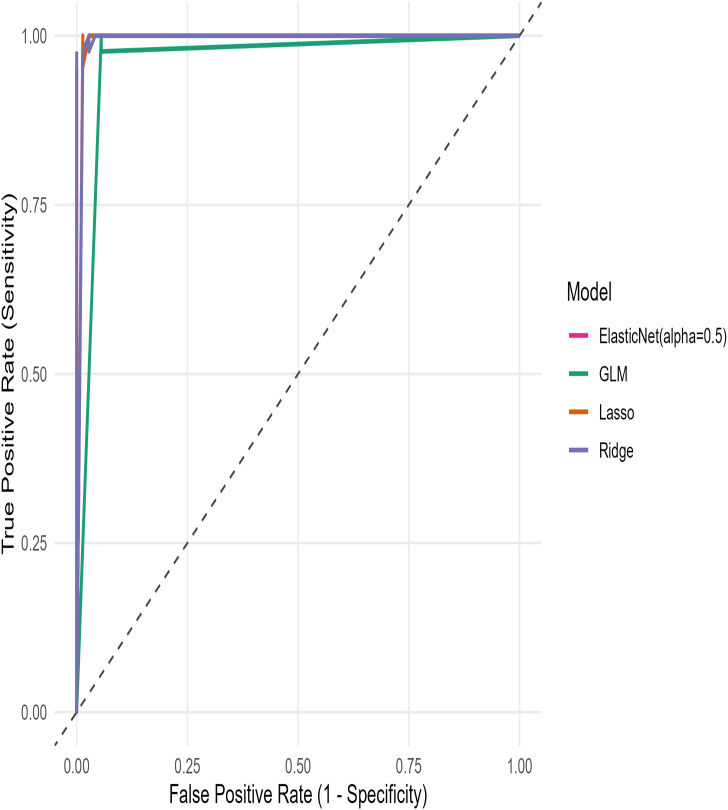
Receiver operating characteristic (ROC) curves comparing all fitted models on the test set.

Fig 1 presents the receiver operating characteristic (ROC) curves for all models evaluated on the test set. While all approaches demonstrate strong discrimination, the ROC curve for the unpenalized logistic regression model lies marginally below those of the penalized models, indicating slightly inferior ranking performance. The ridge, lasso, and elastic-net ROC curves are nearly indistinguishable, reflecting comparable discriminative ability across penalization strategies. Importantly, these results highlight that discrimination alone does not fully characterize model performance in correlated biomedical data; improvements associated with penalization are more apparent in calibration, error balance, and interpretability, which are examined in subsequent sections.

### 5.2. Coefficient shrinkage and sparsity patterns

**Fig 2:**
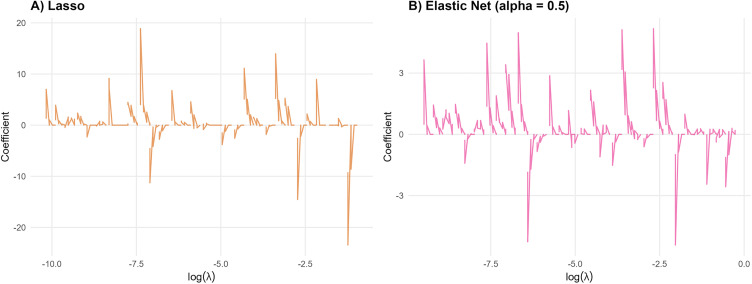
Merged coefficient shrinkage paths for lasso (A) and elastic-net (B) logistic regression models.

Fig 2A illustrates the coefficient shrinkage paths for the lasso logistic regression model as a function of the regularization parameter λ. As λ increases, most coefficient estimates are progressively shrunk toward zero, with many becoming exactly zero, reflecting the sparsity-inducing nature of the lasso penalty. Only a small subset of predictors retains non-zero coefficients across a wide range of λ values, indicating robust contributions to discrimination between malignant and benign tumors. The abrupt entry and exit of coefficients along the solution path further reflect the sensitivity of lasso-based variable selection to the tuning parameter in the presence of correlated predictors. These patterns motivate subsequent evaluation of feature stability across penalization methods.

Fig 2B presents the coefficient shrinkage paths for the elastic-net logistic regression model. Compared with the lasso paths, elastic-net coefficients exhibit smoother and more gradual shrinkage as λ increases, reflecting the stabilizing influence of the $\ell_{\text{2}}$ component of the penalty. Groups of correlated predictors tend to enter and exit the model together, mitigating the abrupt coefficient fluctuations observed under pure lasso penalization. Although sparsity is still achieved at higher values of λ, elastic-net retains a broader set of predictors across a wider range of tuning parameters, suggesting improved robustness of coefficient estimates under multicollinearity.

### 5.3. Feature stability across penalization methods

**Fig 3:**
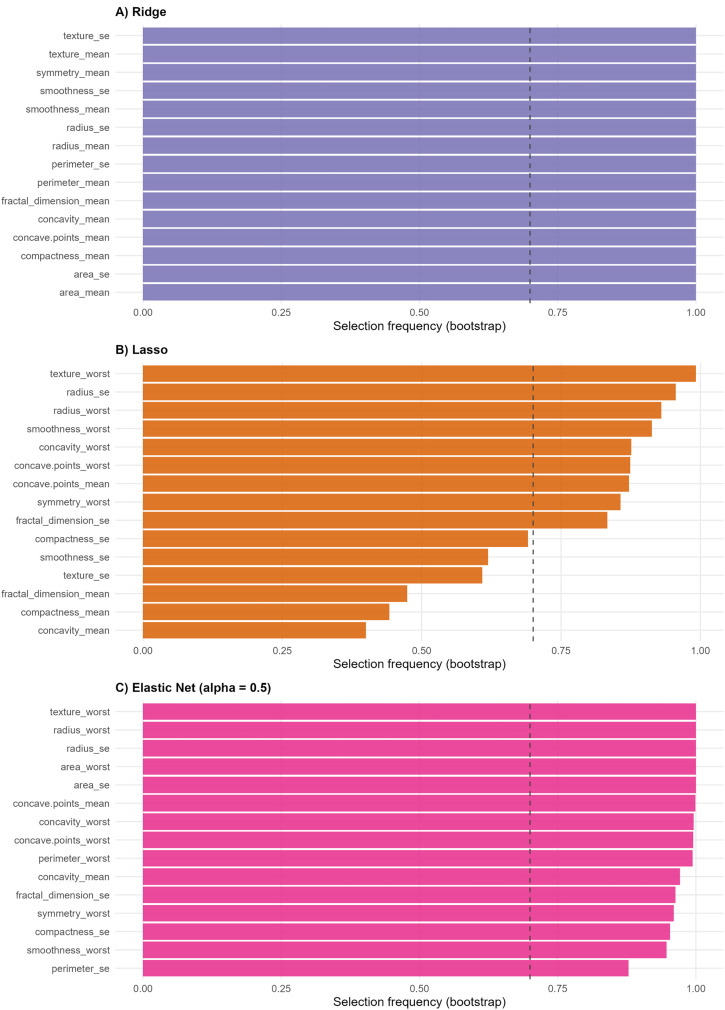
Bootstrap feature stability across penalized logistic regression models (B=1000). Panels A-C display selection frequencies for ridge, lasso, and elastic-net models, respectively. The dashed vertical line indicates the stability threshold $\left(\pi^{*}=0.70\right)$.

Fig 3A displays the bootstrap selection frequencies of the most stable predictors identified by ridge regression. All displayed features exhibit selection frequencies extremely close to one, indicating near-universal inclusion across bootstrap resamples. This behavior reflects the non-sparse nature of ridge penalization, which shrinks coefficients continuously toward zero without performing variable elimination. While this exceptionally high level of stability supports reproducibility of coefficient estimates, it offers limited interpretability in terms of identifying a parsimonious subset of diagnostically relevant predictors.

Fig 3B presents the bootstrap selection frequencies of the most stable predictors identified by lasso regression. In contrast to ridge regression, lasso produces a markedly sparser predictor set, with only a subset exhibiting consistently high selection frequencies above the stability threshold. Variables related to tumor texture, radius, smoothness, symmetry, concavity, and concave points are selected most consistently, indicating robust associations with malignancy. Several additional predictors display moderate selection frequencies, reflecting sensitivity of lasso-based variable selection to sampling variability in the presence of correlated features. These results reinforce the trade-off between sparsity and reproducibility under lasso penalization.

Fig 3C displays the bootstrap selection frequencies of the most stable predictors identified by the elastic-net logistic regression model. Elastic-net retains a broader subset of predictors with uniformly high selection frequencies, with many features approaching perfect selection stability across resamples. Compared with lasso regression, elastic-net demonstrates substantially greater stability while preserving meaningful sparsity relative to ridge regression. The retained predictors largely represent correlated tumor characteristics related to size, shape, and boundary irregularity, consistent with the grouped-selection behavior induced by the combined $\ell_{\text{1}}$ and $\ell_{\text{2}}$ penalty structure. Collectively, these findings indicate that elastic-net regression provides the most favorable compromise among stability, sparsity, and interpretability in this application.

### 5.4. Stable feature sets and interpretability

Table 2 summarizes the stable predictor sets identified by ridge, lasso, and elastic-net logistic regression using a selection-frequency threshold of $\pi^{*}=0.70$. Ridge regression identifies all 30 predictors as stable, reflecting its non-sparse nature and consistent inclusion of correlated variables across bootstrap resamples. While this result indicates maximal stability, it provides limited interpretability for isolating a concise set of diagnostically relevant features, since nearly all predictors are retained regardless of relative importance.

**Table 2:** Stable predictor sets identified by ridge, lasso, and elastic-net logistic regression using a bootstrap selection-frequency threshold of $\pi^{*}=0.70\;\;\left(B=1000\;\;resamples\right)$.

model	n_stable	stable_features
Ridge	30	radius_mean, texture_mean, perimeter_mean, area_mean, smoothness_mean, compactness_mean, concavity_mean, concave.points_mean, symmetry_mean, fractal_dimension_mean, radius_se, texture_se, perimeter_se, area_se, smoothness_se, compactness_se, concavity_se, concave.points_se, symmetry_se, fractal_dimension_se, radius_worst, texture_worst, perimeter_worst, area_worst, smoothness_worst, compactness_worst, concavity_worst, concave.points_worst, symmetry_worst, fractal_dimension_worst
Lasso	9	texture_worst, radius_se, radius_worst, smoothness_worst, concavity_worst, concave.points_worst, concave.points_mean, symmetry_worst, fractal_dimension_se
ElasticNet	22	radius_se, area_se, radius_worst, texture_worst, area_worst, concave.points_mean, concavity_worst, concave.points_worst, perimeter_worst, concavity_mean, fractal_dimension_se, symmetry_worst, compactness_se, smoothness_worst, perimeter_se, compactness_mean, texture_se, smoothness_se, fractal_dimension_worst, symmetry_mean, fractal_dimension_mean, area_mean

In contrast, lasso regression yields a substantially smaller stable set of nine predictors, primarily composed of worst tumor characteristics and a small number of variability-related measures, including texture_worst, radius_worst, smoothness_worst, concavity_worst, concave.points_worst, concave.points_mean, symmetry_worst, radius_se, and fractal_dimension_se. This compact stable set highlights lasso’s ability to produce highly interpretable and parsimonious models, although at the cost of greater sensitivity to sampling variability among correlated predictors.

Elastic-net regression produces an intermediate stable set of 22 predictors, retaining groups of correlated features related to tumor size, shape, texture, and boundary irregularity while excluding less consistently informative variables. This broader yet still selective stable predictor set reflects the grouped-selection behavior induced by the combined $\ell_{\text{1}}$ and $\ell_{\text{2}}$ penalties. Compared with lasso, elastic-net preserves substantially greater stability among correlated predictors, while remaining considerably more interpretable than ridge regression.

Overall, these findings reinforce that the choice of penalty affects not only predictive performance but also the reproducibility and interpretability of selected predictors. Among the models considered, elastic-net provides the most favorable balance among sparsity, feature stability, and interpretability, making it particularly attractive for stability-aware biomedical prediction modeling.

Having established differences in feature stability and model sparsity across penalization methods, the next section evaluates whether these structural differences translate into reliable probability estimates through an assessment of model calibration.

### 5.5. Calibration and probability accuracy

**Fig 4:**
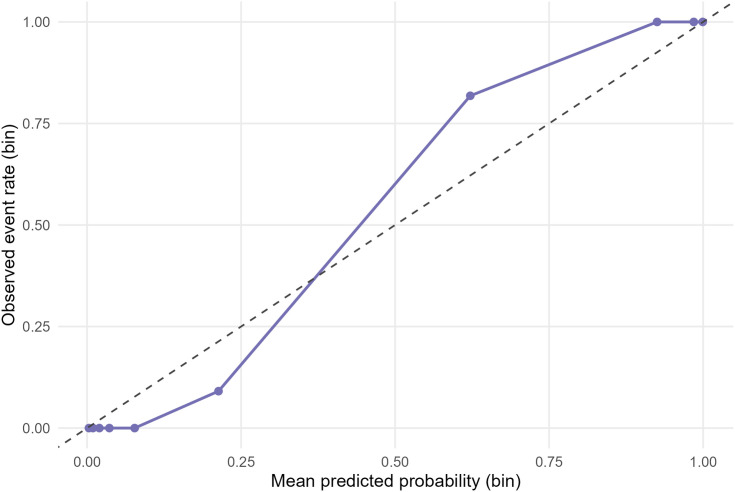
Decile-based calibration plot for the best-performing model selected by AUC.

Fig 4 presents the decile-based calibration plot for the best-performing model selected by AUC, which in this case is ridge logistic regression. The calibration curve closely follows the 45-degree reference line across most probability bins, indicating good agreement between predicted malignancy probabilities and observed event rates. Minor deviations are observed at lower predicted probabilities, where risk is slightly underestimated, while calibration improves at moderate to high probability levels.

These calibration results complement the earlier discrimination and stability findings. Although all penalized models demonstrate near-perfect discriminative ability, meaningful differences emerge in their structural properties: ridge regression provides maximal stability with reliable probability estimates, lasso offers highly parsimonious but less stable feature selection, and elastic-net balances sparsity and reproducibility among correlated predictors. Taken together, these results emphasize that strong discrimination alone is insufficient for clinical modeling and that stability and calibration are critical for developing interpretable, reproducible, and clinically meaningful risk prediction models.

## 6. Discussions

The primary objective of this study was not to introduce a new modeling technique, but rather to demonstrate how stability diagnostics can be systematically integrated into applied penalized regression workflows for biomedical prediction. Using breast cancer risk prediction as a motivating application, we examined feature stability and interpretability in ridge, lasso, and elastic-net logistic regression models within a unified evaluation framework that combines discrimination, calibration, and resampling-based stability assessment [1,10]. Although all penalized models achieved near-perfect discriminative performance on the held-out test set, substantial differences emerged in their feature stability profiles.

Ridge regression consistently retained all predictors across bootstrap resamples, reflecting maximal stability but minimal sparsity. While such behavior supports reproducibility of coefficient estimates, it limits interpretability by failing to isolate a concise subset of diagnostically relevant features. In contrast, lasso regression produced highly sparse models with a relatively small number of stable predictors, enhancing interpretability but exhibiting greater sensitivity to sampling variability, particularly in the presence of correlated predictors [4,5]. Elastic-net regression demonstrated intermediate behavior, retaining correlated predictor groups with consistently high selection frequencies while excluding less informative variables. Notably, the updated bootstrap analysis using B=1000 resamples reinforced the stability patterns observed in the original analysis, with qualitative conclusions remaining unchanged. This consistency strengthens confidence in the robustness of the reported feature stability findings.

These results underscore the importance of assessing feature stability alongside predictive performance in penalized regression modeling. Penalization alone does not guarantee reproducible or interpretable variable selection, especially in biomedical datasets characterized by multicollinearity and moderate sample sizes. Without explicit stability assessment, selected predictors may reflect sampling variability rather than robust signal, thereby limiting interpretability and scientific reproducibility [8]. The elastic-net penalty mitigates these concerns by combining $\ell_{\text{1}}$ and $\ell_{\text{2}}$ regularization, promoting grouped selection among correlated predictors while preserving meaningful sparsity, consistent with theoretical expectations and prior empirical work [5].

From a clinical modeling perspective, feature stability is closely tied to transparency, interpretability, and trust. Models that consistently identify predictors across resampled datasets provide stronger justification for clinical relevance and are more amenable to downstream integration into decision-support systems. Moreover, well-calibrated probability estimates are essential for individualized risk assessment and shared clinical decision-making [1,10,15]. The present findings demonstrate that stability and calibration provide complementary insights beyond discrimination alone and should be considered integral components of model evaluation in medical prediction tasks.

Several limitations warrant consideration. First, the analysis is based on a single, well-studied benchmark dataset, which may limit generalizability to other clinical settings, populations, or data modalities. Although the Wisconsin Diagnostic Breast Cancer dataset provides a valuable methodological benchmark, caution is warranted in extrapolating specific empirical findings beyond this application. Second, external validation using an independent cohort was not performed. While bootstrap-based stability analysis provides insight into reproducibility under resampling, it does not substitute for validation on independent data [1]. Third, observations with missing values were removed during preprocessing using complete-case analysis. While this approach simplifies implementation, it may reduce statistical efficiency and introduce bias when missingness is not completely at random. Future work could investigate multiple-imputation–based stability assessment frameworks to evaluate the robustness of penalized variable selection under incomplete data settings.

Future research may extend this framework to higher-dimensional biomedical applications, including genomic, radiomic, and multi-omics data, where instability in variable selection is often more pronounced. Additional extensions include integration with cost-sensitive or utility-based evaluation frameworks that explicitly account for asymmetric clinical consequences of diagnostic errors, as well as development of formal inferential procedures for stability measures, such as confidence intervals or hypothesis-testing frameworks for selection frequencies, which would further strengthen the role of stability analysis in applied medical modeling [8].

## 7. Conclusion

This study investigated feature stability and interpretability in penalized logistic regression within a stability-aware evaluation framework for biomedical prediction modeling. Although ridge, lasso, and elastic-net logistic regression models achieved similarly strong discriminative performance, they exhibited markedly different stability and sparsity characteristics, demonstrating that predictive accuracy alone is insufficient for comprehensive model evaluation in correlated biomedical data [1,10].

By integrating resampling-based stability assessment with traditional measures of discrimination and calibration, this study provides a more complete framework for evaluating penalized regression models from both predictive and interpretive perspectives. Among the methods considered, elastic-net regression offered the most favorable practical balance between sparsity, feature stability, and interpretability, making it particularly attractive for modeling settings characterized by correlated predictors.

More broadly, these findings emphasize that stability diagnostics should be considered an integral component of applied predictive modeling workflows, especially in biomedical research where reproducibility, transparency, and clinically meaningful interpretation are essential for trustworthy model development and downstream decision support.
